# Supplementary material for: Reaction Time and Visual Memory in Connection to Alcohol Use in Persons with Bipolar Disorder
Source: Brain Sci. 2021 Aug 30;11(9):1154. doi: 10.3390/brainsci11091154 (PMC8467646; doi:10.3390/brainsci11091154)
Supplement: Supplementary file 1 [file brainsci-11-01154-s001.zip › brainsci-1280477-supplementary.pdf]

brain sciences

Supplementary documents

Supplementary Table S1. Association between background factors and hazardous drinking[ψ] in male and female persons with bipolar disorder.

	Male			Female		
	Hazardous drinking			Hazardous drinking		
	No	Yes	p-value	No	Yes	p-value
n	287	179		584	209	
Age	47.06 (13.32)	42.50 (12.06)	<0.001	45.51 (12.82)	41.39 (12.09)	<0.001
Education						
No matriculation examination	190 (66.2)	119 (66.5)	1.000	310 (53.1)	126 (60.3)	0.086
Matriculation examination	97 (33.8)	60 (33.5)		274 (46.9)	83 (39.7)	
Household pattern						
with spouse	107 (37.0)	66 (36.5)	0.981	267 (45.3)	75 (35.9)	0.022
Other	182 (63.0)	115 (63.5)		322 (54.7)	134 (64.1)	
MHI-5						
Depressed	200 (69.7)	131 (73.2)	0.481	415 (71.1)	168 (80.4)	0.011
Non-depressed	87 (30.3)	48 (26.8)		169 (28.9)	41 (19.6)	
Psychotropic medication						
No	15 (5.2)	12 (6.7)	0.645	29 (5.0)	7 (3.3)	0.523
Yes	272 (94.8)	167 (93.3)		554 (94.9)	202 (96.7)	
Missing				1 (0.2)	0 (0.0)	

RT = Reaction time

PAL = Paired association learning

[ψ]AUDIT-C cutoff scores for hazardous drinking were ⩾6 for males and ⩾5 for females

SD = Standard deviation

CI = Confidence interval

[a] Adjusted with age, household pattern, depression (MHI-5) and education

** Analyzed with log-linear regression*

*** Analyzed with linear regression*

**** Analyzed with logistic regression*

Supplementary Table S2. Distribution of RT median, RT SD, PAL first trial memory scores (FTMS) and PAL total errors adjusted in study population.

	Min	1st Qu,	Median	Mean	3rd Qu.	Max
**RT Median**	282.0	385.0	416.0	426.4	454.2	982.5
**RT SD**	17.44	34.97	44.97	55.03	58.15	1069.65
**PAL FTMS**	0.0	7.00	11.00	10.67	14.00	20
**PAL TEA**	0.0	8.00	17.00	23.75	41.00	69

RT = Reaction time
PAL= Paired association learning
FTMS = First trial memory score
TEA = Total error adjusted
SD = Standard deviation

Supplementary Table S3. RT median and RT SD *p*-values for background factors and alcohol related disorder in persons with bipolar disorder.

	Male			***Female***			
	**RT**		**PAL**	**RT**		**PAL**	
	median	SD	FTMS	median	SD	FTMS	Test
Age	<.001	<.001	<.001	<.001	<.001	<.001	Spearman
Education	0.253	0.036	<.001	0.126	0.113	<.001	Point biserial
Alcohol related disorder	0.141	0.340	0.012	0.487	0.325	0.085	Point biserial
MHI-5	0.636	0.125	0.656	0.269	0.109	0.016	Point biserial

RT = Reaction time

PAL = Paired association learning

SD = Standard deviation

Supplementary Table S4. PAL total error adjusted scores for background factors and alcohol use patterns in persons with bipolar disorder.

	Male			***Female***		
	**PAL total errors adjusted**		**p-value**	**PAL total errors adjusted**		**p-value**
	0	1		0	1	
n	268	104		455	221	
Age	47.73 (12.46)	35.92 (10.30)	<0.001	45.85 (12.51)	38.27 (10.17)	<0.001
Education						
No matriculation examination	189 (70.5)	52 (50.0)	<0.001	265 (58.2)	97 (43.9)	0.001
Matriculation examination	79 (29.5)	52 (50.0)		190 (41.8)	124 (56.1)	
Household pattern						
with spouse	108 (40.3)	34 (32.7)	0.216	199 (43.7)	101 (45.7)	0.689
without spouse	160 (59.7)	70 (67.3)		256 (56.3)	120 (54.3)	
MHI-5						
Depressed	184 (68.7)	77 (74.0)	0.372	316 (69.5)	177 (80.1)	0.005
Non-depressed	84 (31.3)	27 (26.0)		139 (30.5)	44 (19.9)	
Psychotropic medication						
No	17 (6.3)	7 (6.7)	1.000	19 (4.2)	15 (6.8)	0.274
Yes	251 (93.7)	97 (93.3)		435 (95.6)	206 (93.2)	
Missing				1 (0.2)	1 (0.0)	

Hazardous drinking						
No	158 (59.0)	63 (60.6)	0.866	341 (74.9)	159 (71.9)	0.459
Yes	110 (41.0)	41 (39.4)		114 (25.1)	62 (28.1)	
Alcohol related disorder						
No	163 (60.8)	76 (73.1)	0.036	330 (72.5)	185 (83.7)	0.002
Yes	105 (39.2)	28 (26.9)		125 (27.5)	36 (16.3)	
PAL = Paired association learning						

Supplementary Table S5. Cohen's d measure of effect.

	Male	Female
**Hazardous drinking**		
RT SD	0.23 (0.04, 0.43)	0.17 (0.02, 0.33)
RT Median	0.30 (0.10, 0.49)	0.28 (0.12, 0.45)
PAL FTMS	-0.05 (-0.26, 0.16)	-0.13 (-0.30, 0.05)
PAL TEA	0.03 (-0.18, 0.24)	-0.07 (-0.24, 0.10)
**Alcohol related disorder**		
RT SD	-0.10 (-0.29, 0.10)	-0.08 (-0.25, 0.08)
RT median	-0.14 (-0.34, 0.05)	-0.06 (-0.22, 0.11)
PAL FTMS	0.27 (0.06, 0.49)	0.15 (-0.02, 0.33)
PAL TEA	0.24 (0.01, 0.44)	0.29 (0.12, 0.47)
RT = Reaction time<br>PAL= Paired association learning<br>FTMS = First trial memory score<br>TEA = Total error adjusted<br>SD = Standard deviation		

Supplementary Table S6. RT median and RT SD for hazardous drinking in persons with bipolar disorder.

		Male			***Female***		
		**Hazardous drinking**			**Hazardous drinking**		
		**0**	**1**	**p-value**	**0**	**1**	**p-value**
RT	Median	440.64 (85.04)	418.84 (51.20)	0.003	427.95 (68.74)	409.81 (50.42)	0.001
	SD	60.79 (58.20)	48.86 (34.75)	0.017	56.94 (61.14)	47.47 (26.49)	0.037
PAL	FTMS	9.83 (5.28)	10.08 (4.93)	0.643	10.93 (4.74)	11.52 (4.52)	0.153
RT = Reaction time<br>*PAL= Paired association learning*<br>*FTMS = First trial memory score*<br>*SD = Standard deviation*							

Supplementary Table S7. Association of reaction time and visual memory with hazardous drinkingψ in bipolar disorder without adjusting depression.

**Five choice reaction time***			
**Median**		**SD**	
**Crude**	**Adjusted[a]**	**Crude**	**Adjusted[a]**

	e^{β} (95% CI)	p-value	e^{β} (95% CI)	p-value	e^{β} (95% CI)	p-value	e^{β} (95% CI)	p-value
**Male**								
Hazardous drinking	**0.76 (0.63, 0.92)**	**0.005**	0.83 (0.69, 1.00)	0.052	**0.80 (0.66, 0.98)**	**0.027**	0.89 (0.74, 1.07)	0.225
**Female**								
Hazardous drinking	**0.77 (0.67, 0.89)**	**<.001**	**0.82 (0.71, 0.95)**	**0.007**	**0.76 (0.65, 0.89)**	**<.001**	**0.84 (0.73, 0.97)**	**0.017**
	**Good performance in PAL**							
	**FTMS****				**TEA*****			
	**Crude**		**Adjusted[a]**		**Crude**		**Adjusted[a]**	
	**β (95% CI)**	**p-value**	**β (95% CI)**	**p-value**	**OR (95% CI)**	**p-value**	**OR (95% CI)**	**p-value**
**Male**								
Hazardous drinking	0.06 (-0.16, 0.27)	0.595	-0.06 (-0.25, 0.13)	0.520	0.96 (0.61, 1.52)	0.875	0.71 (0.42, 1.19)	0.202
**Female**								
Hazardous drinking	0.12 (-0.04, 0.28)	0.147	0.02 (-0.13, 0.17)	0.830	1.16 (0.80, 1.65)	0.434	1.00 (0.68, 1.46)	0.989

RT= Reaction time PAL= Paired association learning SD= Standard deviation CI= Confidence interval
[Ψ]AUDIT-C cutoff scores for hazardous drinking were ≥6 for males and ≥5 for females
[a] Adjusted with age, household pattern and education
* Analyzed with log-linear regression
** Analyzed with linear regression
*** Analyzed with logistic regression

Supplementary Table S8. Association of reaction time and visual memory with alcohol related disorder in bipolar disorder without adjusting depression.

	**Five choice reaction time**				**Five choice reaction time**			
	**Median**				**SD**			
	**Crude**		**Adjusted[a]**		**Crude**		**Adjusted[a]**	
	**e^{β} (95% CI)**	**p-value**	**e^{β} (95% CI)**	**p-value**	**e^{β} (95% CI)**	**p-value**	**e^{β} (95% CI)**	**p-value**
**Male**								
Alcohol related disorder	1.18 (0.97, 1.43)	0.100	1.01 (0.84, 1.23)	0.888	1.13 (0.92, 1.38)	0.236	0.92 (0.76, 1.12)	0.409
**Female**								
Alcohol related disorder	1.05 (0.90, 1.23)	0.518	1.03 (0.88, 1.19)	0.732	1.03 (0.88, 1.20)	0.728	1.00 (0.87, 1.16)	0.958
	**Good performance in PAL**							
	**FTMS****				**TEA*****			
	**Crude**		**Adjusted[a]**		**Crude**		**Adjusted[a]**	
	**β (95% CI)**	**p-value**	**β (95% CI)**	**p-value**	**OR (95% CI)**	**p-value**	**OR (95% CI)**	**p-value**
**Male**								
Alcohol related disorder	**-0.27 (-0.49, -0.05)**	**0.015**	-0.05 (-0.25,0.15)	0.615	**0.60 (0.36, 0.97)**	**0.040**	0.87 (0.50, 1.51)	0.623
**Female**								
Alcohol related disorder	-0.11 (-0.31,0.02)	0.091	-0.09 (-0.24, 0.07)	0.262	**0.51 (0.33, 0.76)**	**0.001**	**0.54 (0.35, 0.83)**	**0.006**

RT = Reaction time
PAL = Paired association learning
SD = Standard deviation
CI = Confidence interval
[a] Adjusted with age, household pattern and education
* Analyzed with log-linear regression
** Analyzed with linear regression
*** Analyzed with logistic regression
